# The first amber caridean shrimp from Mexico reveals the ancient adaptation of the *Palaemon* to the mangrove estuary environment

**DOI:** 10.1038/s41598-019-51218-5

**Published:** 2019-10-29

**Authors:** Bao-Jie Du, Rui Chen, Xin-Zheng Li, Wen-Tao Tao, Wen-Jun Bu, Jin-Hua Xiao, Da-Wei Huang

**Affiliations:** 1 Institute of Entomology, College of Life Sciences, Nankai University, Tianjin, 300071 China; 2 Key Laboratory of Zoological Systematics and Evolution, Institute of Zoology, Chinese Academy of Sciences, Beijing, 100101 China; 3 Institute of Oceanology, Chinese Academy of Sciences, Qingdao, 266071 China

**Keywords:** Palaeontology, Taxonomy, Marine biology

## Abstract

The aquatic and semiaquatic invertebrates in fossiliferous amber have been reported, including taxa in a wide range of the subphylum Crustacea of Arthropoda. However, no caridean shrimp has been discovered so far in the world. The shrimp *Palaemon aestuarius* sp. nov. (Palaemonidae) preserved in amber from Chiapas, Mexico during Early Miocene (ca. 22.8 Ma) represents the first and the oldest amber caridean species. This finding suggests that the genus *Palaemon* has occupied Mexico at least since Early Miocene. In addition, the coexistence of the shrimp, a beetle larva, and a piece of residual leaf in the same amber supports the previous explanations for the Mexican amber depositional environment, in the tide-influenced mangrove estuary region.

## Introduction

Palaemonidae Rafinesque, 1815 is the largest shrimp family within the Caridea, with world-wide distribution^1^. It is now widely believed that it originated from the marine environment in the indo-western Pacific warm waters, and has successfully adapted to non-marine environments, such as estuaries and limnic environments^2–4^.

*Palaemon* Weber, 1795 is the second most species-rich genus besides the *Macrobrachium* Spence Bate, 1868 in the Palaemonidae^4–6^. The 87 extant species of *Palaemon* are found in various habitats, such as marine, brackish and freshwater^7,8^. The genus has a worldwide distribution, and the most reasonable explanation for the distribution is probably due to dispersion and colonization events^9,10^. Reliable fossil records can provide us with evidences for the origin, phylogeny and separation events between these lineages, but available palaeontology materials are extremely scarce most likely due to the aquatic environment and the relatively weak calcification of the exoskeleton of the shrimps^11^. There have been only three definite fossil species included in the *Palaemon*, all preserved in rock impressions^12^, with the earliest known record originating from Lower Cretaceous in Italy^13^. To date, no record of caridean shrimp preserved in amber has been reported.

Here, we first report a *Palaemon* shrimp preserved in Mexican amber which is famous for rich inclusions, such as fungi, flowers, seeds, pollen, leaves, arachnids, insects, vertebrates, and especially Crustaceans^14^. The amber, collected from Simojovel de Allende, Chiapas, southeastern Mexico, most possibly formed by the resin secreted from the *Hymenaea* in Early Miocene^15–17^. Ever since then, amber has deposited along the Gulf of Mexico coast^16^. From Cretaceous to Miocene, the coastal areas, wetland and continental lowlands were often flooded, so some marine organisms failed to return to the sea and had to gradually adapt to estuaries and freshwater environment, which was one of the main reasons for the high species diversity of this region^2^. Tides thus played an important role in the embedding of more ancient aquatic organisms in Mexican amber, in which Decapoda species are rare relative to ostracods, copepods, tanaidaceans, amphipods, and isopods, and only crabs have been reported so far in Mexican amber^18^.

The shrimp in this study represents the first and oldest definite record of the Caridea species preserved in amber all over the world. Meanwhile, it enriches the inclusions biodiversity of Mexican amber, and suggests the distribution of *Palaemon* in southeastern Mexico before Early Miocene. The coexistence of the amber inclusions, a shrimp, a beetle larva and a piece of leaf, extends our understanding on the paleontological depositional environment. It is reconfirmed that the ancient environment of amber deposition in Mexico is a tidal affected mangrove estuary area.

## Results

### Systematic palaeontology

Order Decapoda Latreille, 1802

Suborder Dendrobranchiata Bate, 1888

Infrorder Caridea Dana, 1852

Superfamily Palaemonoidea Rafinesque, 1815

Family Palaemonidae Rafinesque, 1815

Subfamily Palaemoninae Rafinesque, 1815

Genus *Palaemon* Weber, 1795

#### *Palaemon aestuarius* sp. nov

**Figure 1:**
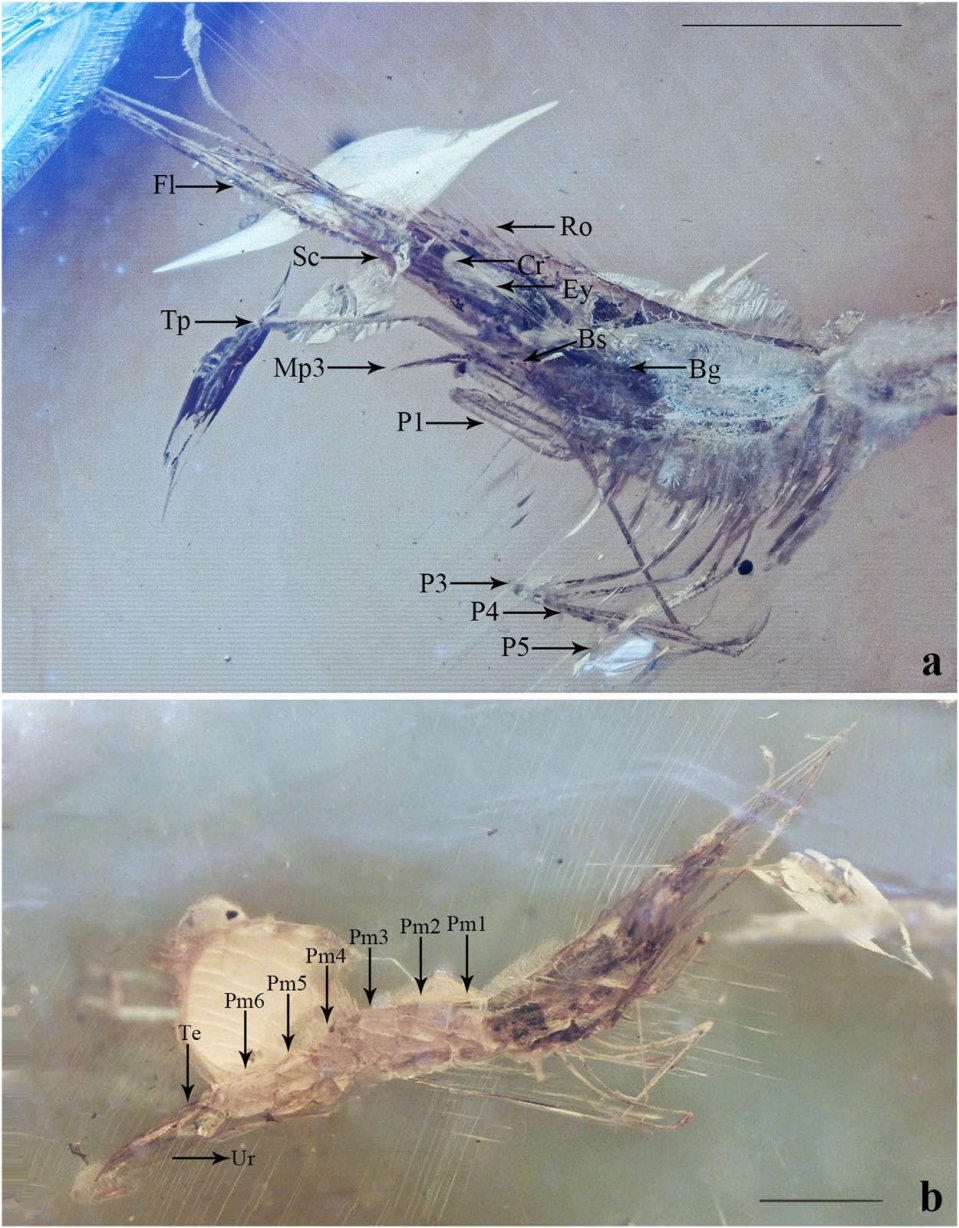
Morphological photographs of *P. aestuarius* sp. nov. (**a**) Detail of cephalothorax in lateral view. (**b**) General view in ateral view. Fl, flagellum; Ro, rostrum; Ey, eyestalk; Co, cornea; Bg, branchiostegal groove; Bs, branchiostegal spine; Sc, scaphocerite; Mp3, third maxillipede; P1, first pereiopod; P3, third pereiopod; P4, forth pereiopod; P5, fifth pereiopod; Pm1, first pleomere; Pm2, second pleomere; Pm3, third pleomere; Pm4, forth pleomere; Pm5, fifth pleomere; Pm6, sixth pleomere; Te, telson; Ur, uropod. Scale bar, 2 mm.

**Figure 2:**
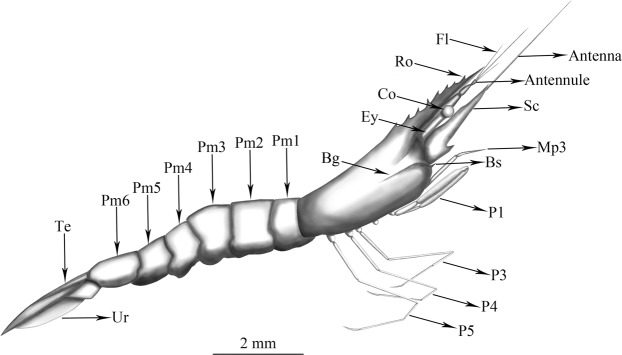
Reconstruction of habitus of *P. aestuarius* sp. nov. (lateral view). The abbreviations represent the same morphological characteristics as the Fig. 1. Scale bar, 2 mm.

Holotype: STJ172. All length measurements are recorded in mm. Total length: 10.3, carapace length: 2.6, rostrum length: 2.2, abdomen length: 4.77, telson length: 1.28 (Figs 1a, b, 2 and S1). The materal deposited in the the Paleo-diary Museum of Natural History, Beijing, China.

Type locality/horizon: Campo La Granja mine, Simojovel de Allende town, Chiapas, southeastern Mexico (Early Miocene, c. 22.8 Ma)^16,19^.

Diagnosis: Rostrum without elevated basal crest, faintly convex in the middle, dorsal margin with nine teeth including one postorbital tooth, basal four teeth evenly distributed, the distance between them greater than the intervals of other teeth. Carapace smooth, branchiostegal groove extended longitudinally backward; branchiostegal spine sharp, situated on anterior margin of cephalon; hepatic spine absent. Length of non-chelate pereiopods increasing gradually from third to fifth; ischium slightly longer than the length of propodus, about 2–3 times as long as carpus, merus longest, dactylus the shortest, apices inwardly hooklike.

Etymology: The specific name comes from the Latinization “estuary” where the shrimp inhabited.

Remarks: *P. aestuarius* sp. nov. resembles *Palaemon vesolensis* Bravi, Coppa, Garassino & Patricelli, 1999, the difference between them in: *P*. *aestuarius* sp. nov. with nine teeth on the dorsal margin, carapace bearing prominent branchiostegal groove; but the *P*. *vesolensis* with seven dorsal teeth, carapace without traces of grooves^20^.

Description: Rostrum long, reaching the distal end of scaphocerite, slightly shorter than the carapace; basal crest absence, dorsal margin nearly straight, faintly convex in the middle; with nine teeth, including one tooth on carapace, large and sharp, tips forward; basal four teeth evenly distributed, the distance between them greater than the intervals of the other teeth.

Eyes well developed, cornea broader than stalk. Antennules triflagellate, scaphocerite slender, about five times as long as wide, outer margin nearly straight, basal area with one long narrow spiny projection, pointed forward. Carapace smooth, hepatic spine absent, without granular process; the branchiostegal groove extended longitudinally backward; branchiostegal spine sharp, situated on anterior margin of cephalon, pointed forward.

The apex of third maxillipede reaching the middle area of scaphocerite. First pereiopod slightly robust, carpus as long as merus, folding inward. The second pereiopod absent. Third pereiopod normal, ischium long, nearly length of propodus; merus longest, about three times as long as carpus; dactylus shortest, with merus ratio 1: 3.6. Forth pereiopod longer than the third pereiopod, ischium slightly longer than the length of propodus, about two times as long as carpus; dactylus shortest, merus longest, with ratio 1: 4.2. Fifth pereiopod longer than the forth pereiopod, ischium nearly equal length of propodus, about two times as long as carpus; dactylus shortest, merus longest, with ratio 1: 4.4.

Abdomen glabrous, with six segments, central uplift, pleurite of the second pleomere covering the pleurites of the first and third; ventral margin of the second pleurites with a central notch, first to third pleurites broadly rounded. Telson conical, 0.6 times as long as sixth abdominal segment; uropods long and narrow triangular, apices sharp, nearly three times the length of the telson.

## Discussion

With the emergence of a variety of aquatic organisms, semiaquatic organisms and insects, the sedimentary paleoenvironment of Mexican amber is generally considered to be a coastal flood-plain suffering from tidal influence in mangrove estuary environment^16,18^. Affected by floods and tides, many ponds and small depressions formed in tidal flat and the surrounding land. Unique environmental conditions make Mexican amber possible to embed various inclusions, including aquatic and terrestrial organisms, particularly abundant crustaceans, relative to other famous amber localities such as Baltic, Dominica, and Myanmar etc.^18,19^. Although numerous aquatic and semi-aquatic groups of amber inclusions have been reported in Mexican amber, such as Copepoda, Ostracoda, Tanaidacea, Amphipoda, Isopoda and Brachyura, there are still more species groups to be discovered and studied^14^. The shrimp presented in this study is the first record of caridean species preserved in amber, adding an important dimension to the inclusion diversity of Mexican amber.

So far, the Palaemonoidea contains nine genera and 17 definite paleontological species, all of which are included in Palaemonidae, and preserved in rock sediments^12,21,22^. Four genera reported entirely in Cenozoic, *Bechleja* Houša, 1957, *Propalaemon* Woodward, 1903, *Pseudocaridinella* Martins-Neto & Mezzalira, 1991, *Micropsalis* Von Meyer, 1859, and we can rule this shrimp out of them by the following morphological features: with triflagellate antennule; having a rostrum with a single tip and lacking hepatic spine; bearing a serrate rostrum on dorsal margin; with a short abdomen and without significant chela on first pereiopod^21,23,24^. There are also four genera recorded in the Mesozoic completely. *Alburnia* Bravi & Garassino, 1998 possesses only one species found in Lower Cretaceous of Italy, which is significantly different from the *P*. *aestuarius* sp. nov. with ten teeth on dorsal rostrum, no traces of branchiostegal groove and branchiostegal spine^25^. *Beurlenia* Martins-Neto & Mezzalira, 1991 has one species preserved in Lower Cretaceous of Brazil, which is distinct from the *P*. *aestuarius* sp. nov. with antennal spine and 14 teeth on dorsal rostrum^26^. *Schmelingia* Schweigert, 2002 has a single species preserved in Upper Jurassic of Germany, which is different from the *P*. *aestuarius* sp. nov. with surface of shell with punctate lines and rostrum with one tooth at apex^27^. *Yongjicaris* Garassino, Yanbin, Schram & Taylor, 2002 has a single species preserved in Lower Cretaceous of China, which is different from the *P*. *aestuarius* sp. nov. with carapace without groove and spine, rostrum without teeth^28^. According to the diagnosis, the shrimp cannot belong to the above genera.

Although the juvenile shrimp in the amber is not well preserved and the most important second pereiopods are missing, we can still classify it into the genus *Palaemon* of Palaemoninae by the following characters: body slender, rostrum armed with non-movable teeth, without coronal projection in basal area, absence of hepatic spine, bearing distinct branchiostegal groove and branchiostegal spine^29^. According to the last two characters, we can separate it from the related genus *Macrobrachium*. In addition, compared to its sister lineage of Pontoniinae, which is strictly marine, with the individuals small and robust, and symbiotic with other marine organisms, Palaemoninae is distributed in a wide salt environment, mainly in shallow sea and freshwater, with individuals relatively thin and living freely. Therefore, we believe that the shrimp should belong to the *Palaemon* of Palaemoninae.

There are three definite fossil species belonging to the *Palaemon*, which are recorded in Europe from Cretaceous and Miocene^12,21,22^. In this study, we report the first *Palaemon* shrimp preserved in amber, which is the first trustworthy fossil *Palaemon* shrimp recorded in Mexico. Therefore, the origin of *Palaemon* can be traced back before the Early Cretaceous and had occupied Mexico at least since Early Miocene.

Seven species of *Palaemon* are known to exist in Mexico. We can distinguish the shrimp from other living species by the following characteristics: *P*. *hobbsi* with five to six teeth on dorsal margin and bearing distinct antennal spine^30^; *P*. *lindsayi* with six or seven dorsal teeth, antennal spine sharp and antennal scale 3.5 times longer than wide^31^; *P*. *mexicanus* with six to seven dorsal teeth, antennal spine sharp and distinct, scaphocerite about three times as long as wide, lateral margin slightly concave^32^; *P*. *mundusnovus* with seven dorsal teeth^33^; *P*. *octaviae* bearing ischium significantly shorter than the length of propodus in the last three pairs of pereiopods^34^; *P*. *paludosus* with six to eight dorsal teeth^35^; *P*. *suttkusi* with five to seven dorsal teeth, scaphocerite almost three times as long as wide, antennal spine strong^36^.

Efficient ability in osmotic pressure regulation enables *Palaemon* shrimps to adapt to the different saline-containing waters. The sedimentary environment of fossil species, recorded from Italy in Cretaceous and Miocene are coastal lagoons, and they share the same prominent feature of lacking branchiostegal groove and branchiostegal spine. This is consistent with the characteristics of the other families completely inhabiting the ocean in Palaemonoidea, including genera in Palaemoninae and all species in Pontoniinae. The shrimp in our study from mangrove estuary environment in Mexico during Early Miocene has branchiostegal groove and branchiostegal spine. Meanwhile, there are seven extant *Palaemon* species in Mexico, distributed in marine sandy mud flats, estuaries and freshwater, and all of them have branchiostegal groove and branchiostegal spine. In conclusion, branchiostegal groove and branchiostegal spine appeared gradually with the marine ancestor continuous invasion from the sea to freshwater, which is consistent with previous studies^37^. Furthermore, in the Early Miocene, there was already *Palaemon* shrimp living in freshwater or estuarine environment, which provided the possibility for further invasion to freshwater.

The most intriguing aspect about the amber is the inclusion of a shrimp together with a beetle larva, which may be a carabid, a staphylinid or a cucujid. The amber is thus so unusual that we try to reconstruct its formation process. During the rainy season in the southeastern Mexico, the weather was hot and humid. *P. aestuarius* sp. nov. lived in mangrove estuary environment, like many other crustaceans found in Mexican amber^16^, tides carrying it with a juvenile coastal beetle rushed to the tidal flat and farther land with the water level rising dramatically. Eventually, they were washed to the edge of a pond surrounded by amber trees, and the shrimp’s athletic ability decreased significantly due to the loss of the second pereiopods during the flushing of the water. More unfortunately, the resin flow was so large that they were wrapped together. Alternative explanation is that the beetle larva clinging to the bark of amber tree was wrapped in flowing resin, and the resin stream captured the shrimp brought by tidal forces at the same time. The resin block continued to grow and flow into the water. When the rainy season passed, the water level of the pond gradually declined and eventually dried up, and the resin was fully solidified and dried. In addition, it is worth noting that we cannot completely rule out the possibility that the shrimp had adapted to freshwater. If so, it is likely that in a flood event, *P. aestuarius* sp. nov. rushed out of the riverbed and fell into a pond. When the pond dried up, the shrimp died, and then the resin flow wrapped it and the beetle larvae. After tens of millions of years of geological action, we finally found them in a piece of amber. In conclusion, the coexistence of the shrimp, a beetle larva, and a piece of residual leaf supports the previous explanations for the Mexican amber depositional environment, a coastal flood-plain affected by tides, rivers and streams, forming a mixed biome of brackishwater, freshwater and terrestrial arthropods^16,38^. Meanwhile, the discovery of *P*. *aestuarius* sp. nov. provides insight into early shrimp evolution and distribution, and sheds light on rich biodiversity in Mexican amber during the Early Miocene.

## Methods

The juvenile shrimp herein preserved in the Campo La Granja mine in Chiapas of Mexico (17°08′48.35″N, 92°42′30.50″W)^16^. The age has been estimated as ca. 22.8 Ma based on strontium isotope ratios (^87^Sr/^86^Sr) measurements in gastropod shells and biostratigraphy information from mollusks^14,16,39^.

The amber is golden brown, translucent with some cracks inside (Fig. S1). In addition to the shrimp, the amber contains a beetle larva and a piece of residual leaf (Figs. S2 to S3). It was cut, grounded and polished, length × width × height about 89 × 89 × 22 mm, and 92.77 g in weight.

The specimen was examined with a LEICA M125 C dissecting microscope. Photographs were taken using a LEICA MC 190 HD fitted to a LEICA M125 C stereomicroscope. Images were stacked with Helicon Focus 6. Figures were prepared in Adobe Photoshop CC and Procreate 4.3.3.

## Supplementary information

The first amber caridean shrimp from Mexico reveals the ancient adaptation of the Palaemon to the mangrove estuary environment
