# Author Correction: The first amber caridean shrimp from Mexico reveals the ancient adaptation of the *Palaemon* to the mangrove estuary environment

**DOI:** 10.1038/s41598-020-59768-9

**Published:** 2020-02-11

**Authors:** Bao-Jie Du, Rui Chen, Xin-Zheng Li, Wen-Tao Tao, Wen-Jun Bu, Jin-Hua Xiao, Da-Wei Huang

**Affiliations:** 1 Institute of Entomology, College of Life Sciences, Nankai University, Tianjin, 300071 China; 2 Key Laboratory of Zoological Systematics and Evolution, Institute of Zoology, Chinese Academy of Sciences, Beijing, 100101 China; 3 Institute of Oceanology, Chinese Academy of Sciences, Qingdao, 266071 China

Correction to: *Scientific Reports* 10.1038/s41598-019-51218-5, published online 29 October 2019

The information in the Methods section of this Article is incomplete.

The fossil was obtained from raw amber; large quantities of rough stones were purchased from songang amber (international) trading market in China, and the sample was found after polishing. The fossil is available to the public at the Paleo-diary Museum of Natural History, and is located on the fourth floor of the Zhongguan Books Building, Haidan, Beijin, China; the code for the specimen is STJ172.

