# Supplementary material for: The first amber caridean shrimp from Mexico reveals the ancient adaptation of the Palaemon to the mangrove estuary environment
Source: Sci Rep. 2019 Oct 29;9:14782. doi: 10.1038/s41598-019-51218-5 (PMC6820862; doi:10.1038/s41598-019-51218-5)
Supplement: Supplementary file 1 — The first amber caridean shrimp from Mexico reveals the ancient adaptation of the Palaemon to the mangrove estuary environment [file 41598_2019_51218_MOESM1_ESM.pdf]

Supplementary information

The first amber caridean shrimp from Mexico reveals the ancient adaptation of the *Palaemon* to the mangrove estuary environment

Bao-Jie Du[1,+], Rui Chen[2,+], Xin-Zheng Li[3], Wen-Tao Tao[1], Wen-Jun Bu[1], Jin-Hua Xiao[1,*], Da-Wei Huang[1,2,*]

[1]Institute of Entomology, College of Life Sciences, Nankai University, Tianjin 300071, China.
[2]Key Laboratory of Zoological Systematics and Evolution, Institute of Zoology, Chinese Academy of Sciences, Beijing 100101, China.
[3]Institute of Oceanology, Chinese Academy of Sciences, Qingdao 266071, China.
[*]Corresponding. huangdw@ioz.ac.cn (D.-W. H.), xiaojh@nankai.edu.cn (J.-H. X.).
[+]these authors contributed equally to this work.

The following Supporting Information is available for this article:

Fig. S1. Overview of the amber. The arrows indicate the position of the inclusions. Sh, shrimp; Bl, beetle larva; Rl, residual leaf. Scale bar, 2 cm.
Fig. S2. Optical photographs of the beetle larva. (**a**) Dorsal view. (**b**) Ventral view. Scale bar, 500 μm.
Fig. S3. Optical photographs of the residual leaf. (**a**)–(**b**) Both sides. Scale bar, 2 mm.

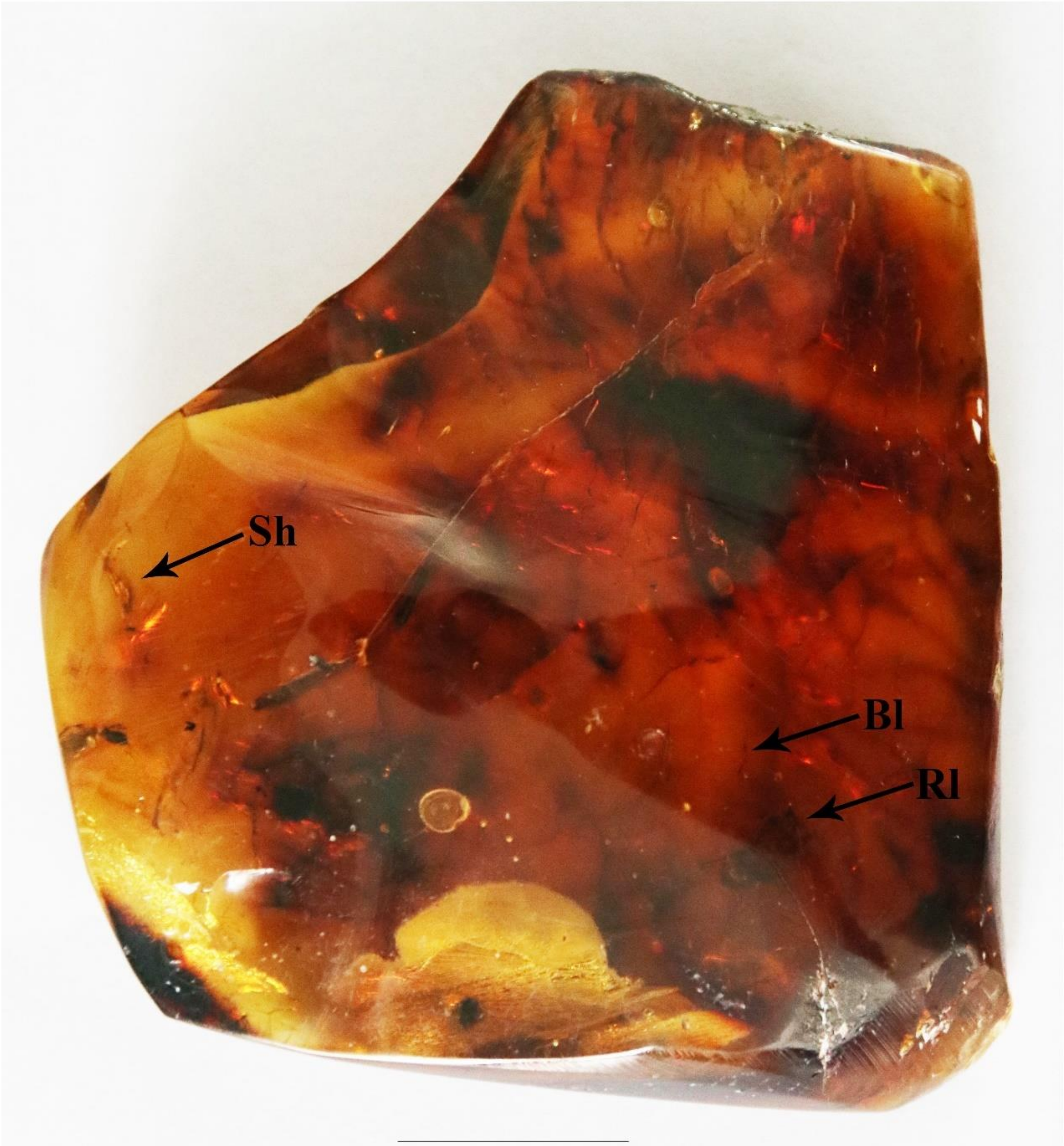
Sh
Bl
Rl

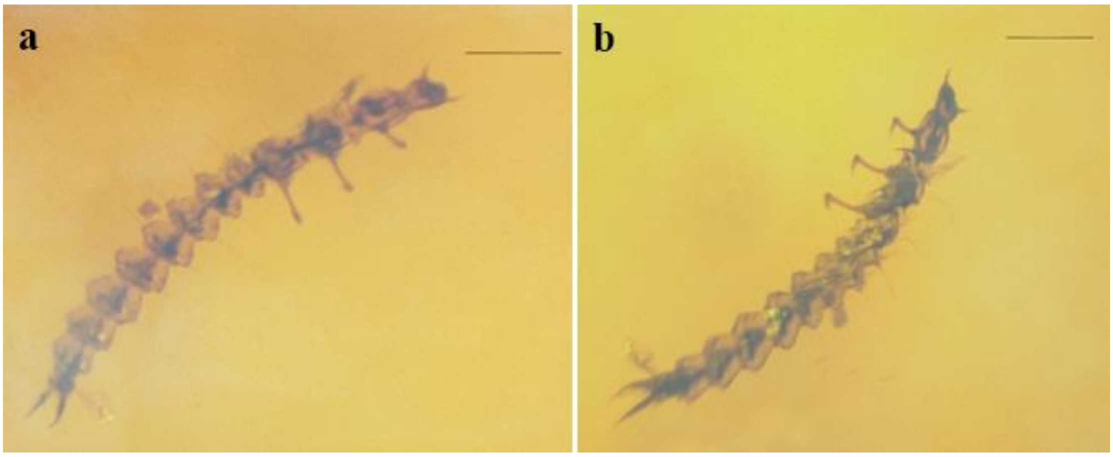
a
b

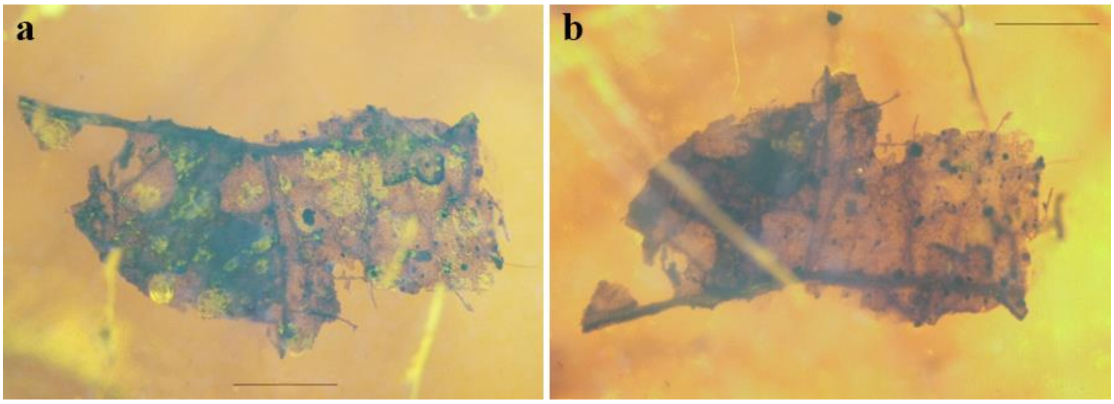
a
b
